# Trends and Predictors of Palliative Therapy Use in Young Adults with Advanced Gastrointestinal Cancer: A National Cancer Database Study

**DOI:** 10.1245/s10434-025-17074-6

**Published:** 2025-03-03

**Authors:** Olivia Monton, Kimberly Kopecky, Andrei Gurau, Orly N. Farber, Elizabeth J. Lilley, Jonathan B. Greer, Fabian M. Johnston

**Affiliations:** 1 Department of Surgery, Johns Hopkins University School of Medicine, Baltimore, MD USA; 2 Department of Epidemiology, Johns Hopkins Bloomberg School of Public Health, Baltimore, MD USA; 3 Department of Surgery, McMaster University, Hamilton, ON Canada; 4 Division of Surgical Oncology, Department of Surgery, University of Alabama at Birmingham Heersink School of Medicine, Birmingham, AL USA; 5 Department of Surgery, Brigham and Women’s Hospital, Boston, MA USA; 6 Center for Surgery and Public Health at Brigham and Women’s Hospital, Boston, MA USA; 7 Department of Psychological Oncology and Palliative Care, Dana-Farber Cancer Institute, Boston, MA USA; 8 Harvard Medical School, Boston, MA USA; 9 Department of Surgery, Massachusetts General Hospital, Boston, MA USA; 10 Department of Surgery, Wake Forest University School of Medicine, Winston-Salem, NC USA

**Keywords:** Palliative care, Palliative therapy, Young adults, Gastrointestinal cancer

## Abstract

**Background:**

Young adults (YAs) with advanced gastrointestinal (GI) cancer have unique care needs, which may be addressed through palliative therapy.

**Objectives:**

The aims of this study were to describe temporal trends and identify predictors of palliative therapy utilization in YAs with advanced GI cancer.

**Methods:**

We conducted a retrospective cohort study using the National Cancer Database. YAs (18–39 years of age) diagnosed with advanced GI cancer from 2004 to 2020 were identified. We performed a trend analysis followed by univariable and multivariable logistic regression analyses.

**Results:**

A total of 43,616 YAs with advanced GI cancer were identified, of whom 3820 (8.76%) were treated with palliative therapy. The proportion of patients who received palliative therapy increased significantly, from 5.33% in 2004 to 12.36% in 2020 (*p*^trend ^< 0.05). Patients of non-White/non-Black race (odds ratio [OR] 1.23, 95% confidence interval [CI] 1.09–1.40), with no insurance (OR 1.35, 95% CI 1.20–1.53), and with a median income of less than $63,000 (OR 1.20, 95% CI 1.08–1.34) were more likely to receive palliative therapy. Multiple comorbidities (OR 1.59, 95% CI 1.24–2.06), stage IV disease (OR 8.28, 95% CI 7.33–9.34), and cancers of the esophagus (OR 2.26, 95% CI 1.88–2.71), liver (OR 2.19, 95% CI 1.88–2.56), pancreas (OR 2.20, 95% CI 1.53–3.16), and biliary tract (OR 2.12, 95% CI 1.54–2.91) were also predictors of palliative therapy utilization.

**Conclusions:**

Palliative therapy utilization in YAs with advanced GI cancer increased significantly over the study period, however major gaps remain in the provision of this care. Further work is needed to understand the barriers to access among YAs.

**Supplementary Information:**

The online version contains supplementary material available at 10.1245/s10434-025-17074-6.

Young adults (YAs), aged 18–39 years, represent an understudied patient population within oncology, with research lagging behind that of other age groups.^1^ This gap raises concerns given the increasing incidence of cancer among YAs in the United States (US).^2^ From 1999 to 2020, there were an estimated 73,954 deaths from gastrointestinal (GI) cancer in YAs in the US alone, translating to an age-adjusted mortality rate of 3.3 per 100,000.^3^ Globally, approximately 131,068 new cases of GI cancer and 79,614 GI cancer-related deaths were reported among YAs in the year 2000, representing approximately 12% of new cancer cases and 25% of cancer-related deaths in this population.^4^

YAs with advanced GI cancer can experience a wide range of symptoms. Patients often present with distressing physical symptoms, including intractable nausea and vomiting, persistent pain, changes in bowel habits, and bleeding. These symptoms may result from gastric outlet obstruction, bowel obstruction, ulceration, and ascites due to uncontrolled tumor growth and direct invasion into surrounding tissues or distant metastases.^5,6^ Beyond the physical manifestations of advanced cancer, YAs have unique psychosocial needs. In a 2021 study, Lidington et al.^7^ evaluated the supportive care needs of YAs with cancer and demonstrated that psychological needs were the most prevalent unmet need, with almost 50% of participants reporting at least one unmet psychological need, including anxiety, feeling down or depressed, feelings of sadness, fears about the cancer spreading or returning, and uncertainty about the future. Although these psychological challenges are not unique to YAs, they may be heightened in this population due to age, life stage, and life transitions.^8,9^

Palliative care is a multimodal medical approach used for patients with serious illness, which focuses on symptom management while addressing the psychosocial and spiritual care needs of patients and their families.^10^ Palliative care can be delivered concurrently with curative-intent treatment or may be the primary focus of care when disease-directed therapy is no longer effective, appropriate, or desired by the patient.^10^ Within cancer care, palliative care may be provided by the primary oncology team or a specialist palliative care team, and often includes some combination of symptom management, psychosocial-spiritual support, advance care planning, and/or procedural interventions, such as surgery, systemic therapy, and radiation therapy.^10^ Early integrated palliative care has been shown to improve quality of life, psychological symptoms, and frequency of care preference discussions, and decrease aggressive end-of-life care in patients with GI cancer.^11,12^ Palliative procedural interventions have been shown to ameliorate symptoms in select patients with GI cancer.^6^ There remains uncertainty about the trends and factors associated with the use of palliative therapy in YAs with advanced GI cancer. The objectives of this study were to describe the trends in palliative therapy use among YAs with advanced GI cancer in the US from 2004 to 2020, and to identify predictors of its use in this patient population.

## Methods

### Data Source

This was a retrospective cohort study using the National Cancer Database (NCDB), a joint project of the American Cancer Society and the Commission on Cancer (CoC) of the American College of Surgeons, which includes prospective data from more than 1500 CoC-accredited cancer programs.^13,14^ The data were obtained from the 2020 NCDB Participant User Files (PUFs) for cancer originating from the digestive system. The 2020 NCDB PUFs contain de-identified data from 2004 to 2020 and are organized by primary site based on the International Classification of Diseases for Oncology, 3rd Edition (ICD-O-3) coding system.^15^ Cancers originating from the following primary sites were included: esophagus (C150–C159), stomach (C160–C169), small intestine (C170–C179), colon (C180–C189), rectosigmoid junction (C199), rectum (C209), anus (C210–C212, C218), liver (C220), pancreas (C250–C259), gallbladder (C239), intrahepatic bile ducts (C221), and other biliary (C240–C249) [Table A in the electronic supplementary material]. This cohort study was granted Institutional Review Board exemption at our institution.

### Patient Population

The study population included YAs (aged 18–39 years) with a diagnosis of advanced (American Joint Committee on Cancer [AJCC] stage III or IV) GI cancer (primary site: esophagus, stomach, small intestine, colon, rectosigmoid junction, rectum, anus, liver, pancreas, gallbladder, biliary tract) from 2004 to 2020. The definition of YAs was based on the 2005 National Cancer Institute and LIVESTRONG Foundation’s Report of the Adolescent and Young Adult Oncology Progress Review Group.^16,17^ Patients with stage I and II disease, missing data on cancer staging, and/or missing data on palliative therapy use were excluded. While older adults were not the primary focus of our study, we conducted a separate analysis including adults aged ≥40 years to compare palliative therapy utilization rates between the two age groups.

### Outcome Variable

The primary outcome was the use of palliative therapy based on the NCDB palliative care variable, which was dichotomized as a binary outcome (palliative therapy, no palliative therapy). This variable captures surgical procedures, radiation therapy, systemic therapy, pain management, or other palliative care, with the goal of alleviating symptoms.^18^ The NCDB uses this variable to distinguish curative-intent treatment from palliative-intent treatment, aimed at alleviating symptoms. This variable, as it is currently characterized by the NCDB, focuses primarily on palliative interventions and pain management, and overlooks other important aspects of palliative care, including specialist palliative care consultation and follow-up, advance care planning, and psychosocial and spiritual support. Although these aspects of palliative care may be included in the ‘other palliative care’ category, the NCDB data dictionary lacks detailed information to confirm this.

### Definition of Study Variables

We included individual-level sociodemographic, medical, and disease characteristics, as well as facility-level characteristics as study variables. Sociodemographic characteristics included age, sex, race, insurance status, median income, and geographic setting. Medical and disease characteristics encompassed medical comorbidities, AJCC cancer stage, and cancer type. Medical comorbidities were measured using the Charlson–Deyo Comorbidity Index (CCI), a weighted score derived from the sum of the scores for comorbid conditions.^19^ Facility-level characteristics included facility type and location. Facility location is currently categorized by the NCDB into nine categories. We collapsed the facility location variable into four categories to improve interpretability.^20^ The four categories included Northeast (New England, Middle Atlantic), South (South Atlantic, East South Central, West South Central), Midwest (East North Central, West North Central), and West (Mountain, Pacific).

### Statistical Analysis

We reported descriptive statistics as means with standard deviations (SDs), medians with interquartile ranges, or frequencies with proportions. Outcomes were compared using t-tests and Pearson’s Chi-square or Fisher’s exact tests for continuous and categorical outcomes, respectively. Using simple linear regression, we described the temporal trends in the use of palliative therapy from 2004 to 2020 overall and by type of palliative therapy (surgery, radiation therapy, systemic therapy, pain management, combination therapy, and other therapy). Univariable logistic regression analyses were conducted to identify associations between any palliative therapy use and sociodemographic, medical, disease, and facility characteristics. Multivariable logistic regression analyses were performed to characterize predictors of palliative therapy use, controlling for select individual- and facility-level characteristics based on clinical and statistical significance. We employed the Hosmer–Lemeshow goodness-of-fit test and the Akaike Information Criterion (AIC) to establish model fit. All analyses were interpreted using a *p*-value of < 0.05. We used STATA version 17.0 (StataCorp LLC, College Station, TX, USA) to analyze the data.

## Results

**Fig. 1:**
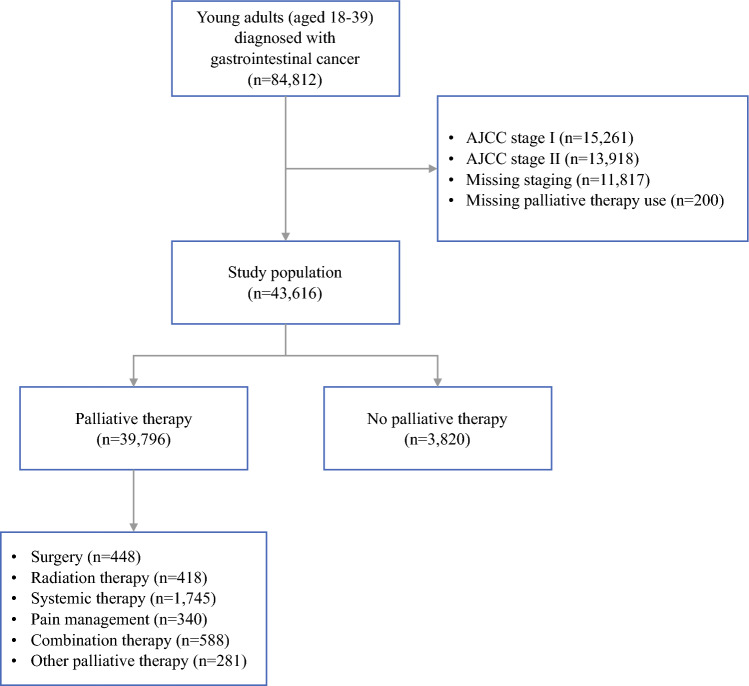
Participant flow diagram. *AJCC* American Joint Committee on Cancer

Our cohort included 43,616 YAs with advanced GI cancer from 2004 to 2020, including cancers of the esophagus (*n *= 1334, 3.06%), stomach (*n *= 5299, 12.15%), small intestine (*n *= 1737, 3.98%), colon (*n *= 17,651, 40.47%), rectum (*n *= 10,419, 23.89%), anus (*n *= 1062, 2.43%), liver (*n *= 2360, 5.41%), pancreas (*n *= 3030, 6.95%), gallbladder (*n *= 275, 0.63%), and biliary tract (*n *= 449, 1.03%). Of the 43,616 patients, only 3820 (8.76%) received palliative therapy, including palliative surgery (*n *= 448, 11.73%), radiation therapy (*n *= 418, 10.94%), systemic therapy (*n *= 1745, 45.68%), pain management (*n *= 340, 8.90%), combination therapy (*n *= 588, 15.39%), and other palliative therapy (*n *= 281, 7.36%). We excluded patients with stage I (*n *= 15,261) and II (*n *= 13,918) disease, and those with missing information on staging (*n *= 11,817) and palliative therapy utilization (*n *= 200). The participant flow diagram is outlined in Fig. 1. Compared with YAs, palliative therapy use was found to be higher among adults aged ≥ 40 years with advanced GI cancer over the study period (12.83% vs. 8.76%).

**Fig. 2:**
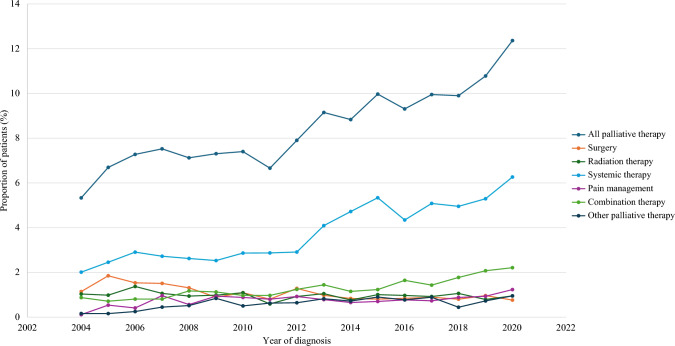
Temporal trends in the use of palliative therapy among young adults with advanced gastrointestinal cancer in the United States from 2004 to 2020

**Fig. 3:**
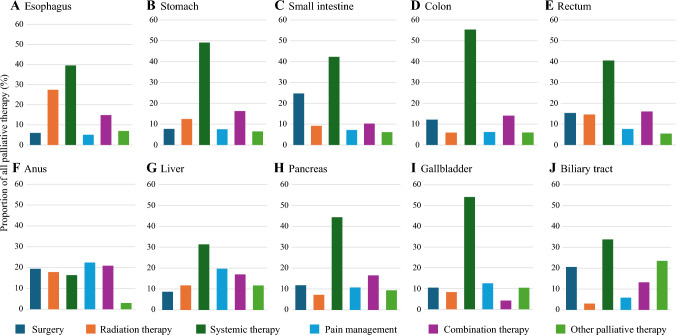
Proportion of patients receiving palliative surgery, radiation therapy, systemic therapy, pain management, combination therapy, and other palliative therapy by cancer type (among patients who received palliative therapy)

Trend analysis revealed that the proportion of YAs with advanced GI cancer receiving palliative therapy significantly increased over the study period, from 5.33% in 2004 to 12.36% in 2020 (*p*^trend^ < 0.05) [Fig. 2]. The proportion of patients receiving systemic therapy, pain management, combination therapy, and other palliative therapy increased significantly over the study period, while the proportion receiving palliative surgery decreased significantly during the same period (*p*^trend^ < 0.05). The most commonly used type of palliative therapy overall and for most cancer types was systemic therapy (esophagus 39.53%, stomach 49.16%, small intestine 42.27%, colon 55.35%, rectum 40.4%, liver 31.34%, pancreas 44.36%, gallbladder 54.17%, and biliary tract 33.82%) [Figs. 2 and 3].

Patients who received palliative therapy were more likely to be male (59.21% vs. 53.32%, *p* < 0.001), of Black race (17.91% vs. 16.21%, *p* < 0.001) or non-White/non-Black race (11.05% vs. 8.77%, *p* < 0.001). They were more likely to be uninsured (12.49% vs. 9.22%, *p* < 0.001) or on Medicare (27.85% vs. 19.57%, *p* < 0.001), live in an urban county (12.85% vs. 10.63%, *p* < 0.001), and have a median income of less than $38,000 (19.61% vs. 17.39%, *p* < 0.001). Those who utilized palliative therapy were also more likely to be treated at an academic/research program (49.42% vs. 44.96%, *p* < 0.001) located in the Midwest (25.00% vs. 23.15%, *p* < 0.001), have one or more comorbidities (CCI 1: 10.39% vs. 7.82%; CCI 2: 1.65% vs. 0.99%; CCI ≥3: 2.72% vs. 1.37%; *p* < 0.001), have stage IV cancer (89.95% vs. 49.28%, *p* < 0.001), and have cancers of the esophagus (5.63% vs. 2.81%, *p* < 0.001), stomach (20.24% vs. 11.37%, *p* < 0.001), liver (8.77% vs. 5.09%, *p* < 0.001), pancreas (13.46% vs. 6.32%, *p* < 0.001), gallbladder (1.26% vs. 0.57%, *p* < 0.001), and biliary tract (1.78% vs. 0.96%, *p* < 0.001) [Table 1].

**Table 1:** Characteristics of young adults with advanced gastrointestinal cancer from 2004 to 2020 [*n* = 43,616]

	No palliative care utilization [*n* = 39,796]	Palliative care utilization [*n* = 3820]	*p*-Value
Sociodemographic characteristics			
Age, years [mean (SD)]	33.64 (4.84)	33.74 (4.75)	0.2225
Year of diagnosis			< 0.001
2004	1740 (4.37)	98 (2.57)	
2005	1715 (4.31)	123 (3.22)	
2006	1824 (4.58)	143 (3.74)	
2007	1833 (4.61)	149 (3.90)	
2008	1984 (4.99)	152 (3.98)	
2009	1980 (4.98)	156 (4.08)	
2010	2201 (5.53)	176 (4.61)	
2011	2213 (5.56)	158 (4.14)	
2012	2307 (5.80)	198 (5.18)	
2013	2334 (5.86)	235 (6.15)	
2014	2529 (6.35)	245 (6.41)	
2015	2699 (6.78)	299 (7.83)	
2016	2717 (6.83)	279 (7.30)	
2017	2834 (7.12)	313 (8.19)	
2018	3059 (7.69)	336 (8.80)	
2019	3054 (7.67)	369 (9.66)	
2020	2773 (6.97)	391 (10.24)	
Sex			< 0.001
Male	21,220 (53.32)	2262 (59.21)	
Female	18,576 (46.68)	1558 (40.79)	
Race			< 0.001
White	29,402 (73.88)	2674 (70.00)	
Black	6449 (16.21)	684 (17.91)	
Other	3492 (8.77)	422 (11.05)	
Missing	453 (1.14)	40 (1.05)	
Insurance status			< 0.001
No insurance	3668 (9.22)	477 (12.49)	
Private	25,504 (64.09)	2016 (52.77)	
Medicare	7787 (19.57)	1064 (27.85)	
Medicaid	1383 (3.48)	152 (3.98)	
Other Government	545 (1.37)	62 (1.62)	
Missing	909 (2.28)	49 (1.28)	
Urbanicity			< 0.001
Metropolitan county	33,292 (83.66)	3155 (82.59)	
Urban county	4232 (10.63)	491 (12.85)	
Rural county	543 (1.36)	58 (1.52)	
Missing	1729 (4.34)	116 (3.04)	
Median income			< 0.001
< $38,000	6922 (17.39)	749 (19.61)	
$38,000–$47,999	8220 (20.66)	877 (22.96)	
$48,000–$62,999	9467 (23.79)	872 (22.83)	
> $63,000	11,130 (27.97)	837 (21.91)	
Missing	4057 (10.19)	485 (12.70)	
Hospital characteristics			
Facility type			< 0.001
Community Cancer Program	2308 (5.80)	187 (4.90)	
Comprehensive Community Cancer Program	12,343 (31.02)	1058 (27.70)	
Academic/Research Program	17,894 (44.96)	1888 (49.42)	
Integrated Network Cancer Program	7251 (18.22)	687 (17.98)	
Facility location			< 0.001
Northeast	7595 (19.08)	869 (22.75)	
South	15,436 (38.79)	1431 (37.46)	
Midwest	9214 (23.15)	955 (25.00)	
West	7551 (18.97)	565 (14.79)	
Medical characteristics			
Charlson–Deyo score (comorbidities)			< 0.001
0	35,744 (89.82)	3256 (85.24)	
1	3114 (7.82)	397 (10.39)	
2	392 (0.99)	63 (1.65)	
≥3	546 (1.37)	104 (2.72)	
Disease characteristics			
NCDB analytic cancer stage			< 0.001
III	20,183 (50.72)	384 (10.05)	
IV	19,613 (49.28)	3436 (89.95)	
Primary cancer site			< 0.001
Esophagus	1119 (2.81)	215 (5.63)	
Stomach	4526 (11.37)	773 (20.24)	
Small intestine	1640 (4.12)	97 (2.54)	
Colon	16,594 (41.70)	1057 (27.67)	
Rectum	9773 (24.56)	646 (16.91)	
Anus	995 (2.50)	67 (1.75)	
Liver	2025 (5.09)	335 (8.77)	
Pancreas	2516 (6.32)	514 (13.46)	
Gallbladder	227 (0.57)	48 (1.26)	
Biliary tract	381 (0.96)	68 (1.78)	

Data are expressed as n (%) unless otherwise specified

SD, standard deviation; NCDB, National Cancer Database

On multivariable analysis, predictors of palliative therapy use were male sex (odds ratio [OR] 1.23, 95% confidence interval [CI] 1.14–1.33) and non-White/non-Black race (OR 1.23, 95% CI 1.09–1.40). Insurance status also played a significant role, with uninsured patients (OR 1.35, 95% CI 1.20–1.53) and Medicare (OR 1.37, 95% CI 1.25–1.51) and Medicaid (OR 1.30, 95% CI 1.06–1.58) recipients having significantly higher odds of palliative therapy use compared to those with private insurance. Living in an urban county (OR 1.15, 95% CI 1.02–1.30) and having a median income of less than $63,000 (<$38,000: OR 1.22, 95% CI 1.08–1.37; $38,000–$47,999: OR 1.27, 95% CI 1.14–1.42; $48,000–$62,999: OR 1.20, 95% CI 1.08–1.34) were also associated with significantly higher odds of receiving palliative therapy. Treatment in an academic/research program or integrated network cancer program increased the odds of palliative therapy use by 25% and 30%, respectively, compared with treatment in a community cancer program (academic/research program: OR 1.25, 95 CI 1.05–1.49; integrated network cancer program: OR 1.30, 95 CI 1.07–1.57). Geographically, higher odds of palliative therapy use were noted in the South (OR 1.32, 95% CI 1.17–1.49), Midwest (OR 1.47, 95% CI 1.30–1.67), and Northeast (OR 1.62, 95% CI 1.43–1.84). Patients with one or more comorbidities (CCI 1: OR 1.30, 95% CI 1.15–1.48; CCI 2: OR 1.43, 95% CI 1.04–1.96; CCI ≥3: OR 1.59, 95% CI 1.24–2.06) also had significantly higher odds of palliative therapy use. The strongest predictor of palliative therapy use was stage IV disease (OR 8.28, 95% CI 7.33–9.34). Compared with colon cancer, cancers of the esophagus (OR 2.26, 95% CI 1.88–2.71), stomach (OR 1.81, 95% CI 1.62–2.02), rectum (OR 1.25, 95% CI 1.11–1.40), anus (OR 1.56, 95% CI 1.14–2.15), liver (OR 2.19, 95% CI 1.88–2.56), pancreas (OR 1.94, 95% CI 1.70–2.20), gallbladder (OR 2.20, 95% CI 1.53–3.16), and biliary tract (OR 2.12, 95% CI 1.54–2.91) were associated with significantly higher odds of palliative therapy use (Table 2).

**Table 2:** Factors associated with palliative therapy use in young adults with advanced gastrointestinal cancer

	Univariable	Multivariable
Sociodemographic characteristics		
Age	1.00 (1.00–1.01)	1.01 (1.00–1.02)
Sex (Ref = Female)		
Male	1.27 (1.19–1.36)	1.23 (1.14–1.33)
Race (Ref = White)		
Black	1.17 (1.07–1.27)	1.06 (0.96–1.18)
Other	1.33 (1.19–1.48)	1.23 (1.09–1.40)
Insurance status (Ref = Private)		
No insurance	1.65 (1.48–1.83)	1.35 (1.20–1.53)
Medicare Medicaid	1.73 (1.60–1.87) 1.39 (1.17–1.65)	1.37 (1.25–1.51) 1.30 (1.06–1.58)
Other Government	1.44 (1.10–1.88)	1.25 (0.91–1.70)
Urbanicity (Ref = Metropolitan county)		
Urban county	1.22 (1.11–1.35)	1.15 (1.02–1.30)
Rural county	1.13 (0.86–1.48)	1.14 (0.84–1.55)
Median income (Ref = >$63,000)		
<$38,000	1.44 (1.30–1.60)	1.22 (1.08–1.37)
$38,000–$47,999	1.42 (1.29–1.57)	1.27 (1.14–1.42)
$48,000–$62,999	1.23 (1.11–1.35)	1.20 (1.08–1.34)
Hospitalization characteristics		
Facility type (Ref = Community Cancer Program)		
Comprehensive Community Cancer Program	1.06 (0.90–1.24)	1.16 (0.97–1.40)
Academic/Research Program	1.30 (1.11–1.52)	1.25 (1.05–1.49)
Integrated Network Cancer Program	1.17 (0.99–1.38)	1.30 (1.07–1.57)
Facility location (Ref = West)		
South	1.24 (1.12–1.37)	1.32 (1.17–1.49)
Midwest	1.39 (1.24–1.54)	1.47 (1.30–1.67)
Northeast	1.53 (1.37–1.71)	1.62 (1.43–1.84)
Medical characteristics		
Charlson–Deyo Score (Ref = 0)		
1	1.40 (1.25–1.56)	1.30 (1.15–1.48)
2	1.76 (1.35–2.31)	1.43 (1.04–1.96)
≥3	2.09 (1.69–2.59)	1.59 (1.24–2.06)
Disease characteristics		
NCDB analytic cancer stage (Ref = Stage III)		
Stage IV	9.21 (8.27–10.25)	8.28 (7.33–9.34)
Cancer type (Ref = Colon)		
Esophagus	3.02 (2.57–3.54)	2.26 (1.88–2.71)
Stomach	2.68 (2.43–2.96)	1.81 (1.62–2.02)
Small intestine	0.93 (0.75–1.15)	1.03 (0.81–1.32)
Rectum	1.04 (0.94–1.15)	1.25 (1.11–1.40)
Anus	1.06 (0.82–1.36)	1.56 (1.14–2.15)
Liver	2.60 (2.28–2.96)	2.19 (1.88–2.56)
Pancreas	3.21 (2.86–3.59)	1.94 (1.70–2.20)
Gallbladder	3.32 (2.42–4.56)	2.20 (1.53–3.16)
Biliary tract	2.80 (2.15–3.65)	2.12 (1.54–2.91)

NCDB, National Cancer Database

## Discussion

This study describes the trends in palliative therapy use among YAs with advanced GI cancer and the factors associated with its use from 2004 to 2020. Our cohort included 43,616 YAs, of whom only 8.76% received palliative therapy. The most commonly used type of palliative therapy was systemic therapy, accounting for 45.68% of all palliative therapy. The proportion of patients receiving palliative therapy increased significantly over the study period, from 5.33% in 2004 to 12.36% in 2020. Sociodemographic predictors of palliative therapy use were male sex, non-White/non-Black race, lack of insurance, Medicare and Medicaid insurance status, urban county residence, and a median income of less than $63,000. Medical and disease characteristics associated with significantly higher odds of palliative therapy use were the presence of one or more comorbidities, stage IV disease, and cancers of the esophagus, stomach, rectum, anus, liver, pancreas, gallbladder, and biliary tract. Facility-level predictors of palliative therapy use included treatment at academic/research programs, integrated network cancer programs, and facilities located in the South, Midwest, and Northeast.

The uptake of palliative therapy among YAs with advanced GI cancer was low, with only 8.76% having received palliative therapy over the study period. Despite a significant increasing trend, the proportion of patients using palliative therapy in 2020 remained low at 12.36%, the highest proportion recorded throughout the study period. This indicates that the vast majority of YAs included in this study did not receive palliative therapy. Several barriers to palliative care use among YAs with cancer are cited in the literature. Chief among them is the difficulty clinicians face when introducing palliative care to YAs. In 2019, Avery et al. conducted semi-structured interviews with medical and radiation oncologists, palliative care physicians, psychiatrists, and advanced practice nurses involved in caring for adolescents and young adults (AYAs) with advanced cancer to explore the challenges of engaging in conversations about palliative care with this population.^21^ Participants described a sense of emotional proximity to the AYA population, compounded by a sense of tragedy, which contributed to their reluctance to engage in discussions about palliative care. Furthermore, they described challenges in helping AYAs and their families to accept and engage with palliative care, as AYAs were perceived as having a decreased ability to accept the reality of having advanced cancer.^21^ The low uptake of palliative therapy outlined in the present study highlights a significant gap in cancer care for this population. Educating clinicians on effective communication and palliative care engagement strategies for YAs and their families is an important next step. Additionally, there is a need for further investigation into the barriers and unique needs of YAs with advanced GI cancer. This would enable the development of targeted interventions and care strategies to ensure this population receives palliative care.

Palliative systemic therapy was the most commonly used therapy overall and by cancer type (excluding anal cancer), accounting for 45.68% of all palliative therapy. As seen in Fig. 2, the increasing trend in palliative therapy overall is likely primarily driven by the increasing use of palliative chemotherapy for patients with advanced cancer.^22,23^ In 2012, an American Society of Clinical Oncology (ASCO) expert panel released guidelines that recommend palliative chemotherapy for solid tumor patients with good performance status.^24^ Although this practice remains controversial,^25–30^ this guideline has led to the widespread use of palliative chemotherapy in patients who have good or moderate performance status who have not responded to curative-intent treatment. Conversely, palliative radiation therapy and surgery are typically reserved for patients with specific symptoms related to complications of advanced cancer, for example bleeding and obstruction.^6^ As a result, the number of patients who are eligible for chemotherapy vastly outnumber those who present with palliative radiation and surgical needs. Despite this, the overall use of palliative interventions remains low, potentially due to a lack of high-quality evidence on the broader benefits of palliative interventions.^6,31^ While clinical practice guidelines advocate for the early integration of palliative care in patients with advanced cancer,^32,33^ similar guidelines for palliative interventions more broadly are lacking. This is partly due to the prioritization of research on disease-directed, curative-intent treatments, which primarily focus on survival outcomes. Additionally, there is significant variability in the ways in which palliative interventions are conceptualized and evaluated in the literature, with many studies failing to measure appropriate patient-centered palliative outcomes.^6,31^ There is a need for high-quality research on the benefits of palliative-intent procedures to inform evidence-based clinical practice guidelines, which may contribute to increased uptake.

Patients with no insurance, Medicare or Medicaid insurance status, and those with a median income of less than $63,000 were more likely to receive palliative therapy. Patients without private insurance or with government-funded insurance, such as Medicare or Medicaid, may have limited access to certain curative treatments due to cost or coverage restrictions and, as a result, may be steered towards palliative options. Insurance status is a known barrier for patients with cancer. In a retrospective cohort study, which evaluated the impact of insurance status on patients with hepatocellular carcinoma, the investigators found that patients with Medicare or Medicaid were less likely to undergo surgical resection and liver transplantation for hepatocellular carcinoma, compared with patients who had private insurance.^34^ Similar trends have been noted in breast, lung, prostate, and colorectal cancer.^35^ Furthermore, patients with lower incomes may face financial barriers in accessing comprehensive cancer care, which may make palliative therapy a more feasible option.^36,37^ The strongest predictor of palliative therapy use was stage IV disease. In patients with stage IV disease, treatment goals are likely to shift from curative- to palliative-intent if disease-directed therapy is no longer effective or appropriate.^10^ Furthermore, patients with stage IV cancer are more likely to experience a higher burden of symptoms and are therefore more likely to benefit from palliative therapy.^38^ Patients with advanced esophageal, gastric, rectal, anal, pancreatic, and hepatobiliary cancers had significantly higher odds of receiving palliative therapy. This is likely due to the aggressive nature of these cancer types when compared with colon cancer.^39^

This study has several limitations. First, our study population consisted solely of YAs aged 18–39 years, excluding adolescents aged 15–18 years. Consequently, our findings do not encompass the broader AYA population, precluding conclusions about the pediatric population or those in transitional care. Second, the palliative care variable in the NCDB differentiates palliative- from curative-intent therapy, and includes surgical interventions, radiation therapy, and systemic therapy. However, the variable also includes pain management and ‘other’ palliative care, where the specific components of ‘other’ palliative care remain unclear. Research in this field tends to differentiate specialist palliative care (consultation and follow-up) from palliative interventions (surgery, radiation therapy, systemic therapy). Therefore, it would be beneficial for the NCDB to capture these elements separately, which would enhance both interpretability and comparability with existing literature. Finally, due to the nature of the database, we were unable to ascertain granular information on disease and treatment characteristics, which would have been useful when interpreting the study findings.

## Conclusions

This study provides an overview of the current state of palliative therapy use among YAs with advanced GI cancer and highlights an important gap in cancer care for this group. Research is needed to further elucidate the barriers to access, which would inform the creation of tailored interventions aimed at enhancing the uptake and utilization of palliative therapy among this population.

## Supplementary Information

Below is the link to the electronic supplementary material.

Supplementary file1 (DOCX 15 KB)

## References

[1] Upshaw NC, Roche A, Gleditsch K, Connelly E, Wasilewski-Masker K, Brock KE. Palliative care considerations and practices for adolescents and young adults with cancer. *Pediatr Blood Cancer*. 2021;68(1):e28781. 33089627 10.1002/pbc.28781

[2] Sung H, Siegel RL, Rosenberg PS, Jemal A. Emerging cancer trends among young adults in the USA: analysis of a population-based cancer registry. *Lancet Public Health*. 2019;4(3):e137–47. 30733056 10.1016/S2468-2667(18)30267-6

[3] Hussaini SMQ, Blackford AL, Sedhom R, Gupta A. Demographic and regional trends of gastrointestinal (GI) cancer mortality in adolescents and young adults (AYA) in the US, 1999–2019. *J Clin Oncol*. 2023;41(4 Suppl):786.

[4] Li J. Digestive cancer incidence and mortality among young adults worldwide in 2020: a population-based study. *World J Gastrointest Oncol*. 2022;14(1):278–94. 35116117 10.4251/wjgo.v14.i1.278 PMC8790416

[5] Harada K, Zhao M, Shanbhag N, Baba H, Ajani JA. Palliative care for advanced gastric cancer. *Expert Rev Anticancer Ther*. 2020;20(7):575–80. 32543938 10.1080/14737140.2020.1781620 PMC7415645

[6] Kopecky K, Monton O, Rosman L, Johnston F. Palliative interventions for patients with advanced gastric cancer: a systematic review. *Chin Clin Oncol*. 2022;11(6):47. 36632980 10.21037/cco-22-102

[7] Lidington E, Darlington AS, Din A, Stanway S, Banerjee S, Szucs Z, et al. Describing unmet supportive care needs among young adults with cancer (25–39 Years) and the relationship with health-related quality of life, psychological distress, and illness cognitions. *J Clin Med*. 2021;10(19):4449. 34640467 10.3390/jcm10194449 PMC8509768

[8] Ferrari A, Thomas D, Franklin ARK, Hayes-Lattin BM, Mascarin M, Van Der Graaf W, et al. Starting an adolescent and young adult program: some success stories and some obstacles to overcome. *J Clin Oncol*. 2010;28(32):4850–7. 20479411 10.1200/JCO.2009.23.8097

[9] Wein S, Pery S, Zer A. Role of palliative care in adolescent and young adult oncology. *J Clin Oncol*. 2010;28(32):4819–24. 20212259 10.1200/JCO.2009.22.4543

[10] Walling AM, Campbell T, Agarwal R, Beck AC, Campbell TC, Carey EC, et al. Palliative Care, Version 1.2024, NCCN clinical practice guidelines in oncology. national comprehensive cancer network; 2024 [cited 10 Jun 2024]. Available at: https://www.nccn.org/professionals/physician_gls/pdf/palliative.pdf

[11] Temel JS, Greer JA, El-Jawahri A, Pirl WF, Park ER, Jackson VA, et al. Effects of early integrated palliative care in patients with lung and GI cancer: a randomized clinical trial. *J Clin Oncol*. 2017;35(8):834–41. 28029308 10.1200/JCO.2016.70.5046 PMC5455686

[12] Merchant SJ, Brogly SB, Goldie C, Booth CM, Nanji S, Patel SV, et al. Palliative care is associated with reduced aggressive end-of-life care in patients with gastrointestinal cancer. *Ann Surg Oncol*. 2018;25(6):1478–87. 29569126 10.1245/s10434-018-6430-9

[13] Mallin K, Browner A, Palis B, Gay G, McCabe R, Nogueira L, et al. Incident cases captured in the national cancer database compared with those in U.S. population based central cancer registries in 2012–2014. *Ann Surg Oncol*. 2019;26(6):1604–12. 30737668 10.1245/s10434-019-07213-1

[14] Cancer Programs American College of Surgeons. Getting Started with the 2020 PUF Data. 2022 [cited 27 Feb 2024]. Available at: https://www.facs.org/media/kezhorot/getting-started-with-the-2020-puf.pdf

[15] Fritz A, Percy C, Jack A, Shanmugaratnam K, Sobin LH, Parkin DM, et al. International classification of diseases for oncology, 3rd ed. World Health Organization; 2000. Available at: https://iris.who.int/handle/10665/42344

[16] Barr RD, Ferrari A, Ries L, Whelan J, Bleyer WA. Cancer in adolescents and young adults: a narrative review of the current status and a view of the future. *JAMA Pediatr*. 2016;170(5):495. 26999630 10.1001/jamapediatrics.2015.4689

[17] National Cancer Institute, LIVESTRONG Young Adult Alliance. Closing the Gap: Research and Care Imperatives for Adolescents and Young Adults with Cancer Report of the Adolescent and Young Adult Oncology Progress Review Group [cited 13 Jun 2024]. Available at: https://www.cancer.gov/types/aya/research/ayao-august-2006.pdf

[18] Cancer Programs American College of Surgeons. National Cancer Database Participant User File: 2020 Data Dictionary. 2022 [cited 27 Feb 2024]. Available at: https://www.facs.org/media/brilfbgu/puf-2020-data-dictionary.pdf

[19] Deyo R. Adapting a clinical comorbidity index for use with ICD-9-CM administrative databases. *J Clin Epidemiol*. 1992;45(6):613–9. 1607900 10.1016/0895-4356(92)90133-8

[20] Chen VW, Portuondo JI, Cooper Z, Massarweh NN. Use of palliative interventions at end of life for advanced gastrointestinal cancer. *Ann Surg Oncol*. 2022;29(12):7281–92. 35947309 10.1245/s10434-022-12342-1

[21] Avery J, Geist A, D’Agostino NM, Kawaguchi SK, Mahtani R, Mazzotta P, et al. “It’s More Difficult…”: clinicians’ experience providing palliative care to adolescents and young adults diagnosed with advanced cancer. *JCO Oncol Pract*. 2020;16(1):e100–8. 31765276 10.1200/JOP.19.00313

[22] Renouf D, Kennecke H, Gill S. Trends in chemotherapy utilization for colorectal cancer. *Clin Colorectal Cancer*. 2008;7(6):386–9. 19036691 10.3816/CCC.2008.n.051

[23] Mehta HB, Vargas GM, Adhikari D, Dimou F, Riall TS. Comparative effectiveness of chemotherapy *vs* resection of the primary tumour as the initial treatment in older patients with Stage IV colorectal cancer. *Colorectal Dis*. 2017;19(6):0210–8. 10.1111/codi.13659 PMC5457355 28304120

[24] Schnipper LE, Smith TJ, Raghavan D, Blayney DW, Ganz PA, Mulvey TM, et al. American society of clinical oncology identifies five key opportunities to improve care and reduce costs: the top five list for oncology. *J Clin Oncol*. 2012;30(14):1715–24. 22493340 10.1200/JCO.2012.42.8375

[25] Braga S. Why do our patients get chemotherapy until the end of life? *Ann Oncol*. 2011;22(11):2345–8. 21917739 10.1093/annonc/mdr416

[26] Kopecky KE, Campbell TC. Renaming palliative cancer therapies: call it what it is. *Oncol*. 2024;29(5):367–8. 10.1093/oncolo/oyae039 PMC11067813 38498046

[27] Meier DE. ‘I don’t want jenny to think i’m abandoning her’: views on overtreatment. *Health Aff*. 2014;33(5):895–8. 10.1377/hlthaff.2013.0517 24799587

[28] Kelly RJ, Smith TJ. Delivering maximum clinical benefit at an affordable price: engaging stakeholders in cancer care. *Lancet Oncol*. 2014;15(3):e112–8. 24534294 10.1016/S1470-2045(13)70578-3

[29] Anders CK, Peppercorn J. Treating in the dark: unanswered questions on costs and benefits of late line therapy for metastatic breast cancer. *Cancer Investig*. 2009;27(1):13–6. 19160108 10.1080/07357900802484944

[30] Prigerson HG, Bao Y, Shah MA, Paulk ME, LeBlanc TW, Schneider BJ, et al. Chemotherapy use, performance status, and quality of life at the end of life. *JAMA Oncol*. 2015;1(6):778–84. 26203912 10.1001/jamaoncol.2015.2378 PMC4828728

[31] Kopecky KE, Monton O, Arbaugh C, Purchla J, Rosman L, Seal S, et al. The language of palliative surgery: a scoping review. *Surg Oncol Insight*. 2024;1(2):100053.

[32] Smith CB, Phillips T, Smith TJ. Using the new ASCO clinical practice guideline for palliative care concurrent with oncology care using the TEAM approach. *Am Soc Clin Oncol Educ Book*. 2017;37:714–23. 28561696 10.1200/EDBK_175474

[33] Ferrell BR, Temel JS, Temin S, Alesi ER, Balboni TA, Basch EM, et al. Integration of palliative care into standard oncology care: american society of clinical oncology clinical practice guideline update. *J Clin Oncol*. 2017;35(1):96–112. 28034065 10.1200/JCO.2016.70.1474

[34] Sobotka LA, Hinton A, Conteh LF. Insurance status impacts treatment for hepatocellular carcinoma. *Ann Hepatol*. 2019;18(3):461–5. 31040093 10.1016/j.aohep.2018.10.001

[35] Stokes SM, Wakeam E, Swords DS, Stringham JR, Varghese TK. Impact of insurance status on receipt of definitive surgical therapy and posttreatment outcomes in early stage lung cancer. *Surgery*. 2018;164(6):1287–93. 30170821 10.1016/j.surg.2018.07.020

[36] Abdelsattar ZM, Hendren S, Wong SL. The impact of health insurance on cancer care in disadvantaged communities. *Cancer*. 2017;123(7):1219–27. 27859019 10.1002/cncr.30431 PMC5360496

[37] Nonzee NJ, Ragas DM, Ha Luu T, Phisuthikul AM, Tom L, Dong X, et al. Delays in cancer care among low-income minorities despite access. *J Women’s Health*. 2015;24(6):506–14. 10.1089/jwh.2014.4998 PMC4490771 26070037

[38] Dillon EC, Meehan A, Li J, Liang SY, Lai S, Colocci N, et al. How, when, and why individuals with stage IV cancer seen in an outpatient setting are referred to palliative care: a mixed methods study. *Support Care Cancer*. 2021;29(2):669–78. 32430601 10.1007/s00520-020-05492-z

[39] Thomas RM, Sobin LH. Gastrointestinal cancer. *Cancer*. 1995;75(S1):154–70. 8000994 10.1002/1097-0142(19950101)75:1+<154::aid-cncr2820751305>3.0.co;2-z

