# Supplementary material for: Trends and Predictors of Palliative Therapy Use in Young Adults with Advanced Gastrointestinal Cancer: A National Cancer Database Study
Source: Ann Surg Oncol. 2025 Mar 3;32(6):4261–71. doi: 10.1245/s10434-025-17074-6 (PMC12049391; doi:10.1245/s10434-025-17074-6)
Supplement: Supplementary file 1 — Supplementary file1 (DOCX 15 KB) [file 10434_2025_17074_MOESM1_ESM.docx]

**Supplementary Material**

**Table A.** Primary site names and codes based on the International Classification of Diseases for Oncology, 3rd Edition (ICD-O-3) coding system.(15)

| **Site name** | **Primary site codes** |
| --- | --- |
| Esophagus | C150-C159 |
| Stomach | C160-C169 |
| Small intestine | C170-C179 |
| Colon | C180-C189 |
| Rectosigmoid junction | C199 |
| Rectum | C209 |
| Anus | C210-C212, C218 |
| Liver | C220 |
| Pancreas | C250-C259 |
| Gallbladder | C239 |
| Intrahepatic bile ducts | C221 |
| Other biliary | C240-C249 |
